# PhosphOrtholog: a web-based tool for cross-species mapping of orthologous protein post-translational modifications

**DOI:** 10.1186/s12864-015-1820-x

**Published:** 2015-08-19

**Authors:** Rima Chaudhuri, Arash Sadrieh, Nolan J. Hoffman, Benjamin L. Parker, Sean J. Humphrey, Jacqueline Stöckli, Adam P. Hill, David E. James, Jean Yee Hwa Yang

**Affiliations:** Charles Perkins Centre, School of Molecular Biosciences, University of Sydney, Camperdown, NSW 2006 Australia; Lowy Packer Building, Victor Chang Cardiac Research Institute, 405 Liverpool Street, Darlinghurst, NSW 2010 Australia; Diabetes and Obesity Program, Garvan Institute of Medical Research, 384 Victoria Street, Darlinghurst, NSW 2010 Australia; School of Mathematics and Statistics, University of Sydney, Camperdown, NSW 2006 Australia; Department of Proteomics and Signal Transduction, Max Planck Institute for Biochemistry, Martinsried, Germany

**Keywords:** Cross-species, Mapping post-translational modification (PTM) sites, Novel phosphorylation site mapping, Web application, Human PTMs, Rat PTMs, Mouse PTMs, Fly PTMs

## Abstract

**Background:**

Most biological processes are influenced by protein post-translational modifications (PTMs). Identifying novel PTM sites in different organisms, including humans and model organisms, has expedited our understanding of key signal transduction mechanisms. However, with increasing availability of deep, quantitative datasets in diverse species, there is a growing need for tools to facilitate cross-species comparison of PTM data. This is particularly important because functionally important modification sites are more likely to be evolutionarily conserved; yet cross-species comparison of PTMs is difficult since they often lie in structurally disordered protein domains. Current tools that address this can only map known PTMs between species based on known orthologous phosphosites, and do not enable the cross-species mapping of newly identified modification sites. Here, we addressed this by developing a web-based software tool, PhosphOrtholog (www.phosphortholog.com) that accurately maps protein modification sites between different species. This facilitates the comparison of datasets derived from multiple species, and should be a valuable tool for the proteomics community.

**Results:**

Here we describe PhosphOrtholog, a web-based application for mapping known and novel orthologous PTM sites from experimental data obtained from different species. PhosphOrtholog is the only generic and automated tool that enables cross-species comparison of large-scale PTM datasets without relying on existing PTM databases. This is achieved through pairwise sequence alignment of orthologous protein residues. To demonstrate its utility we apply it to two sets of human and rat muscle phosphoproteomes generated following insulin and exercise stimulation, respectively, and one publicly available mouse phosphoproteome following cellular stress revealing high mapping and coverage efficiency. Although coverage statistics are dataset dependent, PhosphOrtholog increased the number of cross-species mapped sites in all our example data sets by more than double when compared to those recovered using existing resources such as PhosphoSitePlus.

**Conclusions:**

PhosphOrtholog is the first tool that enables mapping of thousands of novel and known protein phosphorylation sites across species, accessible through an easy-to-use web interface. Identification of conserved PTMs across species from large-scale experimental data increases our knowledgebase of functional PTM sites. Moreover, PhosphOrtholog is generic being applicable to other PTM datasets such as acetylation, ubiquitination and methylation.

**Electronic supplementary material:**

The online version of this article (doi:10.1186/s12864-015-1820-x) contains supplementary material, which is available to authorized users.

## Background

The human genome project revealed surprisingly few protein-coding genes (approximately 20,000) [1], not many more than lower eukaryotes. Protein post-translational modifications (PTMs) such as phosphorylation provide an additional layer of regulation above that of gene expression, creating multi-dimensional cell signaling networks that facilitate the formation of complex development programs and increase the repertoire of inter- and intracellular responses. There are 518 protein kinases in the human genome [2], and it is estimated that these may phosphorylate as many as 1 million different residues under specific conditions [3]. Currently only 118,261 non-redundant human phosphosites are reported in PhosphoSitePlus [4, 5]. Phosphorylation site identification in species such as rat and fly are quite sparse, with only, 14,040 [5] and 10,000 [6], most likely due to much fewer large-scale phosphoproteome studies performed in these organisms. Major challenge of modern cellular biology therefore lie in accurate mapping of PTM sites across the different species without solely relying on already identified sites, quantifying their regulation in response to different stimuli, and assigning protein kinases and biological functions to regulated sites.

Major advances in mass spectrometry (MS) in terms of speed and sensitivity are leading to rapid progress in the global, unbiased identification and quantification of protein phosphorylation of cells and tissues [7]. Indeed large-scale MS-based proteomics studies now routinely identify tens of thousands of phosphorylation sites in different species and biological contexts [8, 9]. In such “discovery”-mode phosphoproteomics studies, MS/MS spectra are obtained and searched against protein sequence databases to identify peptides using a peptide search engine [10–12]. Following peptide identification, any accompanying modifications must be localized to specific amino acid residues and scored, for which several strategies are widely employed [13–15].

Functional PTMs are more likely to be evolutionarily conserved across humans and model organisms such as mice, rats and flies [16]. Consistent with this, conservation is a frequently used criterion by biologists for selecting specific phosphorylation sites of interest for functional characterization. Hence, simplified tools facilitating the mapping of PTMs across different target species would be of particular benefit to the proteomics and cell biology communities. The need to integrate proteomics datasets from multiple species [17–19] has increased recently with the growing availability of large-scale datasets of PTMs from these organisms. However, sites often exist in unstructured flexible domains of proteins in the least conserved domains across species, making mapping on a large scale a substantial challenge. In addition, conservation of sequence positions is particularly poor especially in distantly related organisms such as yeast or flies [17]. Hence, during downstream bioinformatics analysis researchers face challenges when attempting to integrate datasets derived from different species, and even from the same species when differences exist between the exact protein sequence databases used. Specifically, while the exact amino acid position of a modified residue in the primary sequence may in some cases be conserved between mammalian species (i.e. mouse, rat, and human), challenges emerge in directly comparing PTM sites that differ in their amino acid positions.

When the modified amino acid sequence position is not conserved between species, manual searching or aligning the protein sequences to determine the conserved modification site can be performed. PhosphoSitePlus annotates orthologous modification sites between a several organisms including human, mouse, rat, fly and cow [4, 20]. However, it does not allow web-based batch processing of thousands of modification sites from multiple proteomics experiments. While the PhosphoSitePlus database can be downloaded to enable offline mapping between species, this database only contains modification sites identified experimentally in the target species rather than the full repertoire of orthologous residues. For example, the functionally relevant phosphorylation of Acetyl Co-A Carboxylase 1 (ACACA) at S80 in human, S79 in mouse and S79 in rat have all been experimentally identified and are annotated as orthologous modification sites in PhosphoSitePlus. However, in the case of Unc-51-like Kinase 1 (ULK1), the functionally relevant phosphorylation at S758 in human and S757 in mouse have been experimentally identified and are annotated, while the homologous site has not been identified in rat samples and is therefore absent. In this case, comparison of rat phosphoproteomic experiments with another species would exclude this biologically important phosphorylation site, despite its presence in this species. Manual site-by-site query of PTMs sites of ULK1 might elucidate the meta-level information of site conservation across species to the user, but performing similar mapping tasks in a systematic and high-throughput manner is not currently possible.

Several online databases partially address these issues. PhosphoBlast [21] within Phospho.ELM is a database of known phosphorylation sites and allows peptide alignment and position query between human, mouse, rat, fly, yeast and worm. It performs partial alignment of user provided peptide/protein sequences (allowing batch submission) and matches conserved sites in multiple species, but only for those that have been previously reported in the literature. DAPPLE [22] was developed to predict phosphorylation sites in a target species of interest also using experimental evidence of again only known phosphorylation sites obtained from another species, by searching through databases such as PhosphoSitePlus and Phospho.ELM among others. PHOSIDA [23, 24] is another large database that comprises thousands of high-confidence in vivo phosphosites identified by MS-based proteomics in various species. Since PHOSIDA is not a mapping tool but a PTM repository, it can only be used to obtain conservation of known modification site information across species. Hence, with currently available tools, many non-annotated phosphorylation sites that differ in position between species will not be mapped when cross-species data comparisons are made. Exploring the full repertoire of phosphorylation sites identified from MS-based experiments between evolutionarily near and distant target species currently has limitations and is therefore a time-consuming task [19, 25–27]. To this end, we have developed an automated web-based tool, PhosphOrtholog, which allows batch processing and mapping of large species-specific PTM datasets to compare overlap at a site-specific level. To our knowledge, such a tool is not currently available. Our approach therefore advances the proportion of PTMs that can be easily compared across species, enabling accurate mapping of novel, unannotated PTMs. PhosphOrtholog, can retrieve all known sites (such as those reported in PhosphoSitePlus), but its primary function is to identify cross-species conserved PTM sites which are not currently curated. Moreover, PhosphOrtholog is not restricted to use with phosphorylation data, making it more flexible than existing tools, which are limited to one or a select number of PTMs. The confidence of sites mapped with PhosphOrtholog can be inferred from the “E-value” significance score (multiple testing corrected *p*-value; see Methods for details), obtained from the pairwise sequence alignments of orthologous proteins. It requires MS identification site information from *both* species as input to map them to each other.

To demonstrate the utility of PhosphOrtholog, we provide five example data sets (two human-rat pairs and one external mouse phosphoproteomics dataset [28] curated in the PRIDE database [29]), enabling identification of conserved regulatory phosphorylation sites in the insulin and exercise regulated muscle phosphoproteomes, respectively, of human and rat. We also identified the overlap between insulin regulated phosphorylation sites in rat and O-linked β-N-acetylglucosamine (O-GlcNAc) responsive phosphorylation sites in mouse [28] in our third cross-species data pair. We identified 196 regulated conserved phosphorylation sites between human and rat in their insulin stimulated phosphoproteomes, of which 83 were already known and annotated in PhosphoSitePlus, hence, we mapped an additional 113 novel sites which is an increase of 136 % in mapping coverage compared to those retrieved from PhosphoSitePlus [4] alone. In our second dataset, we obtained an increase of 148 % in the mapped coverage of conserved PTMs identified in both species following acute exercise stimuli. In our third example of rat-mouse data, we identified 1315 mapped sites, of which 840 were novel and mapped by PhosphOrtholog, thereby increasing the mapping coverage by 177 %. In all of the above examples, we successfully mapped all sites reported in PhosphoSitePlus, in addition to novel sites. PhosphOrtholog is based on a deterministic algorithm, thus it always produces the same output from a given input. In this study, we only focus on phosphorylation as a representative PTM to illustrate the functionality of PhosphOrtholog. However as mentioned, this application can be extended to map any PTM. Publicly available phosphoproteomics datasets from any two relevant species can be obtained from repositories such as the PRoteomics IDEntifications (PRIDE) database [29], and the overlap of conserved PTMs between these two datasets following some experimental treatment can be easily compared using PhosphOrtholog.

## Implementation and methods

### Data

#### Human-rat dataset 1

Human skeletal muscle insulin-regulated phosphoproteome (1,187 human sites quantified; 551 unique protein accessions): A human skeletal muscle biopsy was obtained from an obese insulin sensitive adult during a hyperinsulinemic-euglycemic clamp (as previously described [30]). Following muscle homogenisation, trypsinisation, fractionation and phosphopeptide enrichment, human muscle phosphopeptides were analysed by LC-MS/MS as described [8]. Following label free MS analysis of human phosphopeptides, RAW MS data were searched and quantified using MaxQuant version 1.3 and the 2011 version of the human International Protein Index (IPI) database to generate the human insulin-regulated phosphoproteome. The IPI identifiers of the human data were converted to their UniProt IDs [31, 32], and only the first UniProt match was retained along with its modification site information.

Rat L6 skeletal muscle myotube insulin-regulated phosphoproteome (10,033 rat sites quantified; 3,050 unique accessions): cells were SILAC labelled [33], and processed as above, enabling quantification of phosphorylation changes in response to insulin (100 nM, 20 min). Four biological replicates (with switching of the SILAC labels) were performed, and analysed by LC-MS/MS as described [8]. RAW MS data was searched and quantified using MaxQuant (version 1.5) and rat FASTA file (October 2013) to generate the rat insulin-regulated phosphoproteome, with UniProt identifiers [31, 32].

#### Human-rat dataset 2

Human and rat skeletal muscles were prepared for MS analysis following exercise stimuli to quantify the number of modified sites in each species as described in detail above (B.L.P., N.J.H., D.E.J., unpublished data). Using the protocol described, we quantified 8,511 human phosphosites and 12,695 rat phosphosites post exercise stimulation.

#### Rat-mouse dataset 3

Mouse phosphoproteome data was obtained from Zhong et al., 2015 [28], the processed Supplementary Table S2 (5,527 phosphorylated S/T/Y sites) from this study was used to parse the “Leading protein”, “Amino acid” and “Position” columns. The mouse data was merged with the rat phosphoproteomic data (10,033 sites) from Dataset 1 to generate Dataset 3.

### Input Requirements for phosphOrtholog

**Fig. 1:**
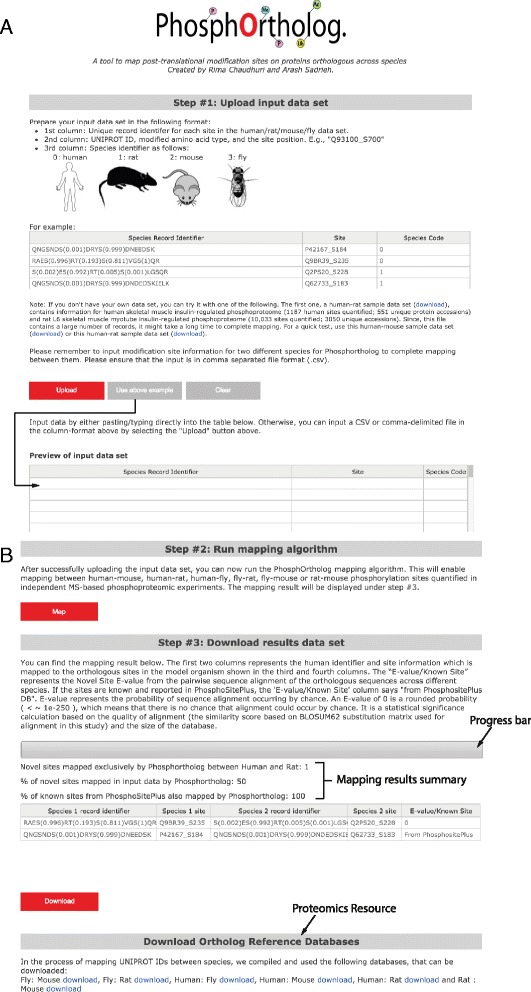
User Interface Snapshots. **a** The instructions for generating the input data format, including each column description is described in “Step #1” in the PhosphOrtholog main page. The input interface also shows an example of the required data format in the table below the text “For example”. The data in the example table can be used as input by clicking the “Use above example” button. Mapping of this data can be completed by clicking “Map”. Input data can also be simply copy-pasted/edited/deleted on the user interface (UI) spreadsheet like with an Excel spreadsheet in the “Preview for input data set” table. Three separate example input files can also be downloaded through the ‘download’ links immediately below the example data table and uploaded to the UI through the “Upload” button. User provided datasets (in comma-delimited format) can be uploaded for mapping *via* the “Upload” button/copy-pasted into the preview input table or typed in. **b** Output Interface: Once mapping is ensued with the ‘Map’ button in “Step # 2”, the progress bar above the output table in “Step # 3” tracks the progress of the mapping function. This will give a rough estimate of how long the job will take to finish for large data sets. The first two columns in the mapped output table indicates the species 1 record identifier and PTM site details which is mapped to the orthologous species 2 site information shown in the third and fourth columns. The last column indicates the E-value significance score from the pairwise sequence alignement of the orthologous proteins. If the PTM site is a known mapped site from PhosphoSitePlus database, then this column reports “From PhosphoSitePlus” instead of a E-value. Once mapping is complete, this bar also reports the number of novel sites mapped by PhosphOrtholog, the percentage of novel sites that could be mapped in the data set and the percentage of known sites from PhosphoSitePlus that could be recovered by PhosphOrtholog

The tool provides a web-based data entry form with 3 columns as a primary input as shown in Table 1 and Fig. 1 that can be copy-pasted into the browser interface, or uploaded as a comma-delimited (.csv) file. It is mandatory to input information for both target species in order to complete mapping between them.

**Table 1:** Example input for PhosphOrtholog

Record identifier	Site	Species code
QNGSNDS(0.001)DRYS(0.999)DNEEDSK	P42167_S184	0
RAES(0.996)RT(0.193)S(0.811)VGS(1)QR	Q9BR39_S235	0
S(0.002)ES(0.992)RT(0.005)S(0.001)LGSQR	Q2PS20_S228	1
QNGSNDS(0.001)DRYS(0.999)DNDEDSKIELK	Q62733_S183	1

Users are required to input data in the format suggested in this table. Information for both species must be entered. The first column must be the record identifier and could be any unique identifier. In the example here, the unique identifiers are the peptide sequences for human and rat PTM sites. The second column represents the Uniprot ID of the species, modified residue type in one letter code and modification site position. An underscore sign must separate the Uniprot ID and site information for each species. The third column represents the species code, human: 0, rat: 1, mouse: 2 and fly: 3. If data for only one species is entered, PhosphOrtholog will return an error asking the user to input data in the correct format

1. The first column should contain a unique identifier or “Record Identifier” for each site in the human/rat/mouse/fly data. This is only required for ‘record keeping’ or maintaining annotation for each input record for example for downstream bioinformatics analysis and is not used in the mapping algorithm. In the example shown in Fig. 1, this column represents output from MaxQuant data analysis software [34] where the protein sequence window around the phosphorylated residue(s) is shown with the probability of detecting the phosphorylation event of a Serine (S)/Threonine (T) or Tyrosine (Y) residue marked within parentheses next to the residue.
2. The second column must contain protein and site information. Required data format: UniProt ID_modified amino acid in one letter code followed by the modification site number with respect to the whole protein (for the UniProt ID provided). For example, Q13085_S23; where Q13085 is the UniProt ID, S is the modified amino acid (Serine) and 23 is the position of the modified residue with respect to the protein Q13085. An underscore sign must separate the UniProt ID and site modification annotation.
3. The third column must contain a species identifier: 0 for human, 1 for rat, 2 for mouse and 3 for fly.
  In summary, input files would resemble the small sample files available in the website which can be downloaded and used as an example input. If only data from one species is used as input, PhosphOrtholog cannot continue mapping since prediction is not one of its functions and will return an error asking for the correct input format. Data can be entered manually, using Copy/Paste functionalities between the web form and other software packages such as Microsoft Excel, or uploaded as comma separated (.csv) files. We have provided the PhosphOrtholog input files for datasets 1 and 3 used in this study (described above) as example input files (Additional file 1 and 2) to guide the users on appropriate input data format for human-rat and rat-mouse data.

### Ortholog reference database generation

An important step in the process involves the creation of six reference databases (DB) for orthologous protein pairs in human-rat, human-mouse, human-fly, rat-mouse, fly-rat and fly-mouse.We created these reference databases through which MS-based experimental data can be screened to obtain a subset of proteins quantified in the experiments with an existing ortholog in the target organism. To generate these, we broadly used four methods and six steps. Databases utilized include HomoloGene (ftp://ftp.ncbi.nlm.nih.gov:/pub/HomoloGene/ July 2014) [35], UniProt knowledgebase (ftp://ftp.uniprot.org/pub/databases/uniprot/current_release/knowledgebase/idmapping/by_organism/ July 2014) [31, 32], InParanoid: Eukaryotic Ortholog Groups [36], and the inter-species mapping tools AnnotationDbi [37] and biomaRt [38, 39] to increase the coverage of proteins within each species. Specifically, the curation procedure followed was as follows:

1. Organism based ID mapping data (selected tab repository) was obtained for human (HUMAN_9606), mouse (MOUSE_10090), rat (RAT_10116) and fly (DROME_7227) from the UniProt knowledgebase.
2. Homologene was then used to map the Entrez Gene IDs between all human proteins obtained above and proteins of each model organism.
3. Corresponding UniProt Accession Name (Uniprot_AC) was obtained for each mapped Entrez Gene ID between the species using the ID mapping file for each organism from 1. In this way we obtained a Uniprot_AC based species:species mapping table.
4. The species-specific Entrez Gene IDs available in R libraries (e.g. org.Hs.eg.db and org.Mm.eg.db) within bioconductor in package AnnotationDbi were used in combination with gene IDs from 2, to map them to the orthologous Entrez gene IDs in each model organism and consequently the Uniprot_AC names using biomaRt. We use ‘ensembl’ marts of each species to achieve this cross-species mapping.
5. InParanoid: ortholog databases were obtained for all pairwise comparisons of the four species of interest in this study, human, rat, mouse and fly from the InParanoid web platform [36]. Records were filtered for > 85 % probability of being an orthologous protein, and if a protein in one species had more than one orthologous protein annotated for the other species, all pairs with an ortholog confidence of > 85 % were retained.
6. Mapping tables from HomoloGene and biomaRt (Uniprot_AC between human and model organisms) were obtained and InParanoid based mapping to obtain the non-redundant orthologous protein pair database between the four species.

In the case of constructing the human-fly DB, an additional resource was used to complete the mapping – the Drosophila RNAi Screening Center (DRSC) [40] derived Integrative Ortholog Prediction Tool [41]. All reviewed UniProt Protein IDs for Drosophila melanogaster were obtained from the UniProt knowledgebase and queried for their human orthologs using the perl script DRSC_orthologs.pl. Mappings were retained if a fly protein was predicted to have a human ortholog by at least one prediction algorithm.

### Architecture

**Fig. 2:**
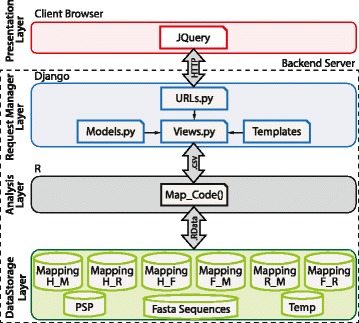
Software architechture. The four layers of the software implementation procedure and the communications between the layers are illustrated. The storage layer shows the six reference ortholog mapping databases where the species are abbreviated by their first letter, for e.g. human by H, rat by R, mouse by M and fly by F. The database of annotated PTM sites obtained from PhosphoSitePlus is represented as PSP

The four different layers of the web-tool are shown in Fig. 2. The presentation layer is implemented using jQuery/HTML5/JSON technology, providing a user interface for the application. This layer is executed on the web browser and communicates to the RequestManager layer through standard HTTP protocol. The RequestManager layer processes the requests received from the presentation layer. RequestManager is implemented using python/Django version 1.5 [42].

The RequestManager layer communicates to the Analysis layer by sending and receiving temporary files in “comma separated” format. The Analysis layer is implemented using the R environment and executes the mapping algorithm described previously. The Analysis layer stores and retrieves information from/to “R data” files and text files located on the backend file system. Mapping_H_R refers to the human-rat ortholog reference database created, Mapping_H_M is human-mouse, human-fly database is named Mapping_H_F, rat-mouse is Mapping_R_M and the fly-mouse and fly-rat databases are named Mapping_F_R and Mapping_F_M respectively. These databases can also be downloaded as comma-separated files from the main web page.

To improve performance we also implemented cache mapping. To achieve this, the web application creates 4,096 bit hash keys from the input string and then associates output to the input using the generated hash key. The web application using FIFO (First In First Out) policy to enforce the cache storage never exceeds the predefined value of 5 GB.

### Algorithm

**Fig. 3:**
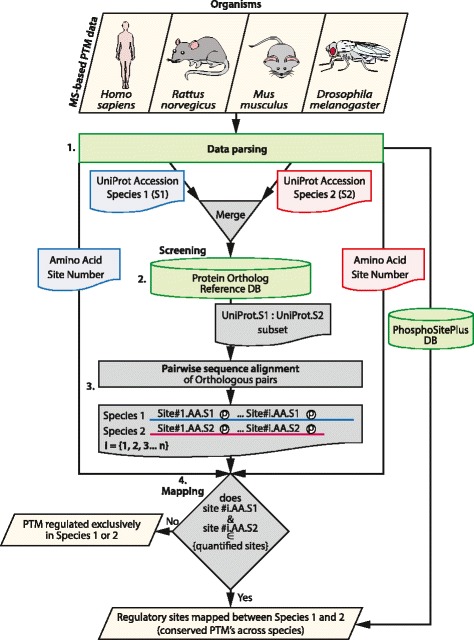
The algorithmic workflow. The schematic representation of the algorithm is depicted through the flowchart. The four stages through which the input data is analyzed to return the mapped sites are displayed

PhosphOrtholog comprises four major processing components, summarized in Fig. 3. The first step involves processing RAW mass spectrometry data using software such as MaxQuant [34] or Proteome Discoverer (ThermoFisher Scientific, CA, USA). The output should include a list of UniProt identifiers, modified residues and their positions (with respect to the UniProt identifier). Input data requires the pre-determined format as described above.

The next step involves screening the input data through the in-house created ortholog reference databases (database creation details described below) to obtain subsets of MS quantified proteins from two independent experiments (in two separate species) that have known orthologous proteins.

Concurrently, we screen the input data through the phosphorylation_site_dataset (obtained from PhosphoSitePlus [4], July 2014, and abbreviated to ‘PSP’ DB for brevity here) to retrieve all known mappings between the two species of interest as curated in PhosphoSitePlus. In summary, if proteins from two species were both previously identified and reported as orthologous in PhosphoSitePlus, we retrieve their orthologous site map or SITE_GRP_ID from PSP and annotate these sites as “From PhosphoSitePlus” which are later output in the mapping results table. This step is similar to the function of DAPPLE [22] and enables assessment of mapping performance by comparing the mapped coverage of PhosphOrtholog to those obtained from PhosphoSitePlus.

Next, we perform global pairwise sequence alignment [43] between orthologous protein pairs using the BLOSUM62 [44–46] substitution matrix, with a gapOpening of 10, gapExtension of 0.5 using Biostrings [43]. The protein sequences for each organism are retrieved from the complete organism-specific proteome FASTA sequence files (UniProt Knowledgebase, January 2015). A raw similarity score (S) is calculated between the pairwise alignments of the two orthologous proteins using BLOSUM62. Next, we convert this raw score to a bit score (S’) using the formula: S' = (lambda*S - ln *K*)/(ln 2), where *K* and *lambda* are statistical parameters dependent upon the scoring system and the background amino acid frequencies of the sequences being compared. We use the estimated values of *lambda* of 0.25 and K of 0.035 [47] from maximum likelihood estimate methods for BLOSUM62 substitution matrix. We then calculate a *p*-value indicating the probability of the alignment (S’) occurring by chance or P(x > S’) = 2^-S’^. The expected value or *E*-value, which is a multiple testing corrected *p*-value is subsequently estimated (*E* = m * n * *p*), where m is the length of the query sequence and n is the length of the database or search space. In this way, we obtain a statistical significance score for each pairwise sequence alignment between orthologous proteins from different species.

Finally, we scan this sequence alignment using modified residue site numbers of one species from the input data (species 1) as the reference to identify the aligned amino acid residues and their positions in the target species (species 2). If a match occurs in the residue type and aligned position in species 2, we consider this as a match and the UniProt ID, residue and site number are retained as a modified site that was successfully mapped between the species.

Overall, the mapping performed is a deterministic process. Input PTMs are mapped to each other based on the match of sequence positions and types (after the global pairwise alignment). There is either a match of residue type and modification site number between the two species compared or not, and therefore there are no random elements involved in the generation of the output data from a given input.

## Results

Here we present PhosphOrtholog, a web-based tool providing cross-species mapping functionality for novel and known phosphorylation and other PTM sites quantified in independent MS-based experiments. PhosphOrtholog allows site-specific mapping between orthologous proteins in human-mouse, human-rat, human-fly, rat-mouse, fly-mouse and fly-rat, allowing batch queries. The web-tool can be accessed from www.phosphortholog.com using any typical web browser (except Internet Explorer). Two novel data sets have been used in this study to exemplify the performance of PhosphOrtholog when compared to PhosphoSitePlus. We find that PhosphOrtholog increases the coverage of conserved phosphorylation sites between human-rat MS experiments by 136 % and 148 % in the two example human-rat datasets and maps novel MS-based modification sites seamlessly between target species.

PhosphOrtholog is the only tool that allows users to map two large-scale phosphoproteomics data and download novel PTM matches between the two target species without relying on *a priori* knowledge of PTM sites. PhosphOrtholog allows batch processing which enables users to map large numbers of sites simultaneously. We present the key features of this web-tool, algorithm and performance of PhosphOrtholog in the following sections.

### Enhanced flexibility through multiple interfaces of PhosphOrtholog: Web-tool, virtual box and R source code

#### 1) Web Tool

##### User Interface

In Fig. 1, the key features of the input and output interface of PhosphOrtholog are highlighted. The correctly formatted user provided input data can be entered through the user interface *via* copy-paste functionality into the “Preview of input data set” table, or alternatively uploaded as a comma-delimited (.csv) file through the “Upload” button (Fig. 1a). Example input data is also provided on the web page, which can be easily copied into the preview-input-table by clicking the “Use above example” button. We also provide three additional data sets (a) human-rat insulin stimulated phosphoproteomics data from the example study presented in this article (b) a subset of (a) which is a small human-rat dataset and (c) a human-mouse data set that can be downloaded through the “download” link and uploaded into the web browser to illustrate the required format of input data and the process of batch file upload. An example of the required input data format can also be found in Table 1.

Once the input data is uploaded and displayed in the input table, the user should click on the “Map” button and the system returns the results on the screen in the output table below in Step # 3 which can be copy-pasted or downloaded as a comma separated file by clicking the “Download” button. The first four output columns will have the same format as the first two input columns; PTM site information of species 1 (first two columns) followed by the PTM information of species 2 (last two columns). The fifth column of the output table reports the E-value confidence score, (which is a probability of sequence alignment occurring by chance) if the cross-species site mapped is not reported in PhosphoSitePlus. If the mapping was previously known, it returns “From PhosphoSitePlus” in the fifth column. The alignment is critical because the accurate determination of orthologous modification sites (position and residue type) is entirely dependent on the pairwise sequence alignment quality. As the system maps the data associated with orthologous protein phosphorylation sites between the target organisms, the progress of mapping large data sets can be assessed through a progress bar above the output columns in Step # 3, which refreshes every 5 s to accurately reflect the mapping progress, as shown in Fig. 1b. Once the mapping is complete, below the progress bar we also report three summary counts: (a) the number of novel sites mapped by PhosphOrtholog in the dataset; (b) the percentage of data that could not have been mapped without PhosphOrtholog i.e. percentage of novel sites mapped in input data; (c) the recovery of known orthologous phosphosites (sites annotated in PhosphoSitePlus for that data set) by PhosphOrtholog.

This easy to use point and click web-based application is primarily designed for the biological research community that is expected to apply the tool to its research without back-end modifications or extensions.

##### Architecture

As highlighted in Fig. 2, a multi-tier architecture was used to build this system [20]. The general software architecture of the PhosphOrtholog webtool is segregated into four distinct layers: 1) Presentation layer, 2) RequestManager layer, 3) Analysis layer, and 4) Data storage layer. The implementation details of each layer are provided in the Methods section. The Presentation layer of the tool defines the creation of the user interface for the application. Since multiple users can access PhosphOrtholog simultaneously, the RequestManager layer has the ability to process multiple requests concurrently. The Analysis layer is implemented in R programming environment and is responsible for the execution of the mapping algorithm, and the Data Storage layer refers to the back-end databases used for data retrieval and query jobs by the R code.

Since most analysis functions implemented in R require a similar workflow of processing input/output data, the software architecture we propose here can be modified to host any other R analysis script. Using the architecture detailed in Fig. 2, we provide the following advantages compared to traditional methods of only deploying R source code.

1. Accessibility: The users can access the system from any connected device with a modern browser ranging from smart phones, tablets to desktop PC.
2. Simplicity: The graphical user interface is simple to use compared with running commands through an R terminal window.
3. Decoupling backend and frontend: The decoupling of backend system from frontend in this client/server architecture will allow computational hungry analysis routines to be executed on high performance computing servers.
4. Simplifying upgrades and release version management: The analyze layer can be upgraded on the backend server whereas the client will use the exact same version of the application, rather than distributing many versions of R scripts between all the users.
5. Zero-install: The users of this architecture can run the application without the requirement of installing R framework and all the required packages.

##### Cache mapping

Since our web application is hosted in the cloud, we implemented a basic caching mechanism to minimize CPU utilization by reusing results that have been previously calculated. This greatly reduces the mapping time required for large data sets.

#### 2) Virtual box

To simplify the process of creating a “test and development environment”, necessary to extend and/or validate our webtool, we created a pre-build environment as a virtual machine, which can be downloaded from (http://goo.gl/hDfJbi). This Virtual Machine will allow users to modify the source code and/or add additional data files without performing all the necessary installation and configuration. The virtual machine was tested on Win7.0/8.1, Ubuntu 14.04 Linux and Mac OS 10.9 operating systems.

#### 3) R Source code

The algorithm behind the R code is explained in the Methods section and Fig. 3. To enhance accessibility and flexibility of this tool to the statistical bioinformatics community, enabling customization or addition of additional functional enhancements, the stand-alone R source code will be made available to interested parties upon request.

**Fig. 4:**
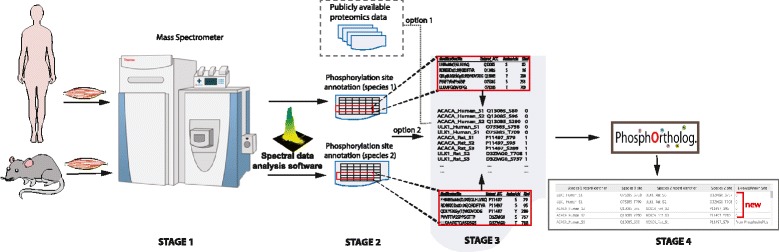
Role of PhosphOrtholog in the MS-based PTM data analysis pipeline. Illustration of the broadly divided four stages of MS-based PTM experiments, in Stage 1, sample extraction and preparation tasks are conducted from human and rat muscle tissues for the MS-based phosphoproteomics experiment. Stage 2 marks the raw spectral data analysis to generate peptide and protein annotations along with intensity measures for the PTMs induced by the experimental design in each species. In Stage 3, output from Stage 2 is parsed to extract information such as the leading Uniprot ID (‘Uniprot_ACC’), modified amino acid type (‘AminoAcid’) and modification site number (‘Site#’) from each species and concatenated in the desired input format for PhosphOrtholog mapping. We showcase the PTM examples for proteins ULK1 (2 sites in human and rat) and ACACA (3 sites in human and rat) here; column ‘ModificationSite’ indicates the peptide sequence with the identified PTM site and the probability of particular amino acids being phosphorylated by the number within the parenthesis. In Stage 4, the sites mapped by PhosphOrtholog are obtained, which are either annotated as newly mapped with a calculated E-value (4 out of 5 input sites were not mapped before, identified with E-value of 0) or with “From PhosphoSitePlus” if the mapping was previously known (mapping between human ACACA site S80 and rat ACACA site S79 is annotated in PhosphoSitePlus database)

In Fig. 4, we depict the role of PhosphOrtholog in the phosphoproteomic data analysis pipeline. We divided the pipeline in four stages; we define Stage 1 as the process of sample extraction and preparation for the mass spectrometry based experiments. The resulting spectra are then pre-processed and evaluated by software such as MaxQuant [34] through multiple steps. In Stage 2, the software generated outputs are obtained which contains protein and modification site level annotations along with quantitative intensity measures from each experiment in Stage 1, these are often large spreadsheets with data in the form of multi-dimensional matrices. In Stage 3, appropriate PTM site annotation columns (Uniprot ID, modified amino acid type and number) are parsed from these output files from each species and merged together in the desired input format for PhosphOrtholog. At this stage, any publicly available proteomics data can similarly be parsed and used as input to PhosphOrtholog. In the final step, PhosphOrtholog maps common sites and produces results, which indicates the newly mapped sites by a calculated E-value, whereas previously known sites are marked by “From PhosphoSitePlus”. We have illustrated this workflow using two example proteins (ULK1 and ACACA) from the human and rat phosphoproteomes in Fig. 4.

### Cross-species protein ortholog reference databases are a proteomics resource

An important component of the algorithm described above includes the creation of the protein orthologous reference databases between human, rat, mouse and fly. The details of the DB creation are provided in the Methods section. In summary, using a six step procedure, we created 6 reference databases including a human-rat reference ortholog DB with 20,530 unique rat UniProt protein IDs that map to their human orthologs, a human-fly DB with 10,476 protein pairs, human-mouse ortholog DB with 25,243 records, rat-mouse ortholog DB with 24,928 protein pairs, fly-rat with 16,072 records and fly-mouse with 16,526 orthologous proteins. These ortholog databases can be downloaded and used by the community. Availability of these databases will substantially reduce analysis time for bioinformaticians working on similar proteomics-based cross-species mapping projects in the future.

### PhosphOrtholog maps novel phosphorylation sites between human-rat and rat-mouse MS experiments and increases coverage of conserved PTMs across species

Here we show that by using PhosphOrtholog we increased the coverage of mapped orthologous PTM sites between two human and rat phosphoproteomics datasets by 136 and 148 %, respectively, when compared with annotated sites. In a third example, we compare the phosphoproteomes of O-GlcNAc responsive mouse fibroblasts and insulin-stimulated rat muscle to increase the mapping coverage by 177 %. In order to gauge the performance of PhosphOrtholog, we employed the PhosphoSitePlus database and used it as a benchmark. Our evaluation criteria is based on (i) inclusion of annotated sites (from PhosphoSitePlus) and (ii) percentage increase in coverage due to identification of novel conserved phosphorylation sites across the two species under consideration by PhosphOrtholog. PhosphoSitePlus has a common identifier called the ‘SITE_GROUP_ID’, enabling easy mapping between known/quantified target species modification sites; these data can be downloaded and mined. However, it should be noted that if a site is experimentally identified in only one species and its orthologous site in the other species is not identified in any previous experimental study, this orthologous protein and site match information cannot be obtained from this database.

#### Performance in dataset 1

**Fig. 5:**
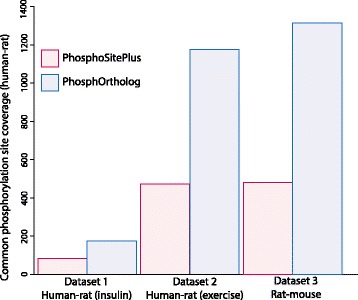
Increased coverage of common sites. Shows the utility and efficiency of PhosphOrtholog compared to PhosphoSitePlus for three example datasets comprising human, rat and mouse phosphoproteomes. The coverage of conserved sites identified by PhosphOrtholog when compared to PhosphoSitePlus was increased by 136 % (from 83 annotated sites in PhosphoSitePlus to 196 mapped sites, an additional 113 novel orthologous PTM site matches) in dataset 1 and by 148 % (from 473 to 1174 mapped sites, an increase of 701 novel site matches) in dataset 2 and by 177 % (from 475 to 1315 sites, thereby adding 840 novel sites matches) in dataset 3

PhosphoSitePlus contained information for 51 % of the quantified human sites and 14 % of the rat sites. Only 83 sites could be mapped between the two datasets through the Site_Group_ID from PhosphoSitePlus whereas using PhosphOrtholog we were able to map 196 sites between the rat and human data. As shown in Fig. 5 PhosphOrtholog enabled mapping of an additional 113 sites, thereby increasing the coverage of mapped sites by more than double (136 %). This example dataset is provided for download on the PhosphOrtholog website and included as Additional file 1 enabling users to submit it as a test file to increase familiarity with the tool.

#### Performance in dataset 2

The PhosphoSitePlus database contained 58 % and 14 % of the human and rat data sets, respectively, that were previously identified phosphosites. However, only 473 phosphosites were annotated to be conserved between human and rat in PhosphositePlus. Fig. 5 summarizes the utility of using PhosphOrtholog for this dataset; we identified 1,174 common phosphosites between the two species, increasing the conserved site overlap by 148 %. PhosphOrtholog added 60 % of the common sites, and the remaining 40 % were already annotated in PhosphoSitePlus. PhosphOrtholog also successfully mapped all known sites reported in PhosphoSitePlus, thus reporting a “known site enrichment” rate of 100 %.

#### Performance in dataset 3

We identified 1315 commonly phosphorylated sites between publicly available mouse phosphoproteome data from Zhong et al., 2015 [28], our in-house generated insulin-stimulated rat phosphoproteome data (rat data from Dataset 1). PhosphoSitePlus contained information about 55 % and 14 % of the quantified mouse and rat sites, respectively. Of the 1315 sites mapped between the two species, 840 of them were previously un-annotated, while 475 sites (36 %) were already curated by PhosphoSitePlus. In summary, we increased the repertoire of known phosphorylated sites between these rat and mouse phosphoproteomes by 177 %. The PhosphOrtholog input file for this example dataset has been provided here as Additional file 2.

Site-specific phosphorylation data for rat is quite sparse; hence, these novel orthologous site mappings are a valuable source of conserved phosphorylation sites for this species and can be easily added to PTM resources such as PhosphoSitePlus to advance coverage of such cross-species conserved sites.

## Discussion

In this study, we present PhosphOrtholog, a web-based tool, which allows mapping of both novel and annotated PTM sites across species for large MS-based phosphoproteomics datasets. We show that by using PhosphOrtholog we increased the coverage of new PTM sites in our three example cases by 136, 148 % and 177 %, respectively, when compared to sites already annotated in PhosphoSitePlus. The PhosphOrtholog algorithm is based on a deterministic approach, where a match is reported only if a match between amino acid type and position occurs between the two aligned sequences. The accuracy of this mapping is dependent on the pairwise sequence alignment of the sequences, which is reflected by the E-value score (see Methods) representing the statistical significance of each alignment. A reported E-value of 0 is a rounded probability (< ~1e-250), which means that there is essentially no chance that alignment could occur by chance. Since PhosphOrtholog is not a prediction algorithm but a mapping tool, common metrics of evaluating performance of predictive tools such as false positive rate or a receiver operating characteristic curves (ROC curve) are not applicable here; this is because PhosphOrtholog is designed to only map PTM sites that have been experimentally identified in two independent cross-species MS experiments. The feature that differentiates it from other mapping tools is its ability to map between hundreds of newly identified PTM sites in different species identified by MS-based proteomics studies (and thereby compute overlap of conserved sites between the two species data compared), enabling mapping of unknown or novel sites or PTM types.

As described, this tool is equally suited to mapping of other PTMs such as acetylation, methylation and ubiquitination. Biologists and bioinformaticians can easily apply this tool in their research due to its user-friendly interface, and the data generated by PhosphOrtholog provides researchers with a repository of orthologous sites modified between target species under similar experimental perturbations.

The in-house generated dual species phosphoproteomics datasets presented in this study are novel and facilitated the development and testing of this tool. Additional datasets can be downloaded by the user from repositories such as PRIDE [29] and mapped to site-specific PTM data of another organism easily using PhosphOrtholog. With such large-scale PTM datasets becoming increasingly available the utility of PhosphOrtholog for comparing PTM site conservation across species will further increase, facilitating the identification of novel conserved functional residues, an important goal of systems biology research.

## Conclusions

Bioinformatics platforms that simplify the integration of large-scale PTM data from different species will be invaluable for the proteomics field and will save both laboratory scientists and bioinformaticians considerable time and effort. Furthermore, the transition of global proteomic analysis of animal models to a targeted clinical investigation involving human samples is often of high importance, since this enables translation of laboratory-based research to the clinic. However, this progression is hindered by numerous factors including target protein orthology. In the wake of increasing proteomics-based systems biology research, the need for tools to facilitate data integration across species is increasing. Current tools such as PhosphoSitePlus, DAPPLE and Phospho.ELM allow cross-species mapping, but rely on known/annotated sites already reported in the literature. No easily accessible tool exists that can compute overlap of known and novel conserved PTM sites across species on a large-scale.

Here, we present a novel web-based automated tool that simplifies mapping of user provided PTM sites from large MS-based experiments between different species. The utility of this tool becomes apparent when we consider the number of additional novel conserved and regulated sites we effectively map between experiments in different species under similar stimuli when compared to those obtained from already available PTM resources. PhosphOrtholog allows users to query novel orthologous phosphorylation and other PTM sites between species, and is not solely reliant on annotation from previously published modification data. Moreover it enables users to batch-process large data sets, simplifying the identification of commonly regulated sites between cross-species experiments with an easy-to-use web-based interface.

## Availability and requirements

- Project name: PhosphOrtholog
- Project home page: www.PhosphOrtholog.com
- Operating system(s): Platform independent. Compatible with any modern web browser except Internet Explorer (we tested it on Firefox v30.0, Chrome v36.0, Safari v6.1)
- Programming language: jQuery/HTML5, Python/Django, R
- Other requirements: A VirtualBox installation (for expansion and modification)
- License: GPL
- Any restrictions to use by non-academics: None

## Ethics and consent

Dataset 1 (Human-rat L6 muscle cells, insulin): Ethical approval for the human study was obtained from the St Vincent’s Hospital Health Research Ethics Committee, Sydney. Informed written consent was obtained from all participants. Rat experiments were performed on *in vitro* L6 muscle cells.

Dataset 2 (Human-rat, exercise): The human study was approved by the regional ethics committee in Denmark (Journal number: H-1-2012-006) and carried out in accordance with the Declaration of Helsinki II. Written informed consent was obtained from each subject. All rat experiments were approved by the Animal Ethics Committee of The University of Melbourne and were conducted in accordance with the Australian code of practice for the care and use of animals for scientific purposes as stipulated by the National Health and Medical Research Council (Australia).

## Additional files

Additional file 1:
**PhosphOrtholog input file for Dataset 1.** (CSV 505 kb) Additional file 2:
**PhosphOrtholog input file for Dataset 3.** (CSV 153 kb)

## Abbreviations

(PTM): Post-translational modifications
(MS): Mass-spectrometry
(DB): Database
(PSP DB): Phosphorylation_site_dataset
